# Supplementary material for: Benthic grazing in a eutrophic river: cascading effects of zoobenthivorous fish mask direct effects of herbivorous fish
Source: PeerJ. 2018 Feb 14;6:e4381. doi: 10.7717/peerj.4381 (PMC5816581; doi:10.7717/peerj.4381)
Supplement: Supplemental Information 1 — This file contains a description of the preliminary experiments that we performed to find the optimal construction of fish exlosure tiles and to test whether the electrical electric exclusion effectively prevents fish from foraging. [file peerj-06-4381-s001.pdf]

Preliminary experiments

We performed two consecutive 24-h experiments with five adult specimens of nase taken from the river Nister in an artificial indoor stream channel (2.6 m × 0.9 m × 0.5 m) filled with water also taken from the river Nister (520 L, conductivity 347 µs cm^{-1}). The photoperiod during the experiments was 15:9 (light:dark) h. Fish were not fed for three days prior to the experiments.

In the first experiment, we exposed two different electrified exclosure tiles in the stream channel to find the optimal construction: one with two aluminium insulators (variant A) and the other with two aluminium insulators and two additional steel insulators (variant B). The second experiment aimed to test whether a) electric exclusion effectively prevents fish from foraging and b) fish feed on the periphyton growing on the non-electrified control tile. Thus, in the second experiment, we exposed one electrified exclosure tile and one non-electrified control tile in the stream channel. Prior to the experiments, the tiles were preconditioned in the river Nister for two weeks to grow periphyton, which was used as bait during the experiments. The exclosure tiles were connected to a fence charger (compact B400, Electra Landtechnik GmbH, Vöhl, Germany, 0.3 J output energy) that emitted electric pulses every 1.22 s for 2.04 ms. Electric pulses were measured using an oscilloscope (TEK 2245A, American Tektronix, Beaverton, US). During the experiments, fish contacts with tiles were filmed with a webcam (320 × 320 pixel resolution) that was installed above the stream channel. The number of fish contacts with tiles and contact durations were analysed using VLC media player (version 2.2.1). This analysis was only possible for the day period.

In the first experiment, we observed one fish contact of 8 s with the variant A tile and three contacts with a total duration of 17 s with the variant B tile. As both constructions of electric exclosures seemed to be similarly effective in preventing fish from foraging, we used the more simple construction (variant A) in the second experiment. In the second experiment, there were considerably more fish contacts with the non-electrified control tile than with the electrified exclosure tile (control: 86 contacts, 45.3 min total duration; exclosure: 5 contacts, 1.4 min total duration). During the experiment, the fish intensively grazed periphyton on the non-electrified control tile and almost completely removed the periphyton from the tile, whereas there were no traces of feeding on the electrified exclosure tile at the end of the experiment.
